# Changes in SCD gene DNA methylation after bariatric surgery in morbidly obese patients are associated with free fatty acids

**DOI:** 10.1038/srep46292

**Published:** 2017-04-10

**Authors:** Sonsoles Morcillo, Gracia Mª Martín-Núñez, Sara García-Serrano, Carolina Gutierrez-Repiso, Francisca Rodriguez-Pacheco, Sergio Valdes, Montserrat Gonzalo, Gemma Rojo-Martinez, Francisco J. Moreno-Ruiz, Alberto Rodriguez-Cañete, Francisco Tinahones, Eduardo García-Fuentes

**Affiliations:** 1 Unidad de Gestión Clínica de Endocrinología y Nutrición, Instituto de Investigación Biomédica de Málaga (IBIMA), Hospital Clínico Virgen de la Victoria, Málaga, Spain; 2 CIBER Fisiopatología de la Obesidad y Nutrición (CIBEROBN), Instituto de Salud Carlos III, Málaga, Spain; 3 Unidad de Gestión Clínica de Endocrinología y Nutrición, Instituto de Investigación Biomédica de Málaga (IBIMA), Hospital Regional Universitario, Málaga, Spain; 4 CIBER de Diabetes y Enfermedades Metabólicas Asociadas (CIBERDEM), Instituto de Salud Carlos III, Málaga, Spain; 5 Unidad de Gestión Clínica de Cirugía General, Digestiva y Trasplantes, Instituto de Investigación Biomédica de Málaga (IBIMA), Hospital Regional Universitario, Málaga, Spain; 6 Unidad de Gestión Clínica de Aparato Digestivo, Instituto de Investigación Biomédica de Málaga (IBIMA), Hospital Clínico Virgen de la Victoria, Málaga, Spain

## Abstract

Stearoyl CoA Desaturase-1 (SCD) is considered as playing an important role in the explanation of obesity. The aim of this study was to evaluate whether the DNA methylation SCD gene promoter is associated with the metabolic improvement in morbidly obese patients after bariatric surgery. The study included 120 subjects with morbid obesity who underwent a laparoscopic Roux-en Y gastric by-pass (RYGB) and a control group of 30 obese subjects with a similar body mass index (BMI) to that found in morbidly obese subjects six months after RYGB. Fasting blood samples were obtained before and at six months after RYGB. DNA methylation was measured by pyrosequencing technology. DNA methylation levels of the SCD gene promoter were lower in morbidly obese subjects before bariatric surgery but increased after RYGB to levels similar to those found in the control group. Changes of DNA methylation SCD gene were associated with the changes of free fatty acids levels (r = −0.442, p = 0.006) and HOMA-IR (r = −0.249, p = 0.035) after surgery. RYGB produces an increase in the low SCD methylation promoter levels found in morbidly obese subjects. This change of SCD methylation levels is associated with changes in FFA and HOMA-IR.

Stearoyl CoA desaturase-1 (SCD) has been proposed as playing a vital role in the explanation of obesity in Mediterranean countries^1^. SCD is an endoplasmic reticulum-bound enzyme that transform different saturated fatty acids into monounsaturated fatty acids (MUFA)^2^. Animal and human studies have revealed the association between SCD and obesity and insulin resistance^3^,^4^. Mice with a disruption in the SCD gene are resistant to diet induced weight gain and have increased insulin sensitivity when compared with wild-type controls^5^. However, a study in mice found that SCD deficiency worsens the diabetes in obese mice^6^. However, total SCD deficiency represents an extreme phenotype that may not easily be compared with human physiology.

Bariatric surgery has proved to be the most effective treatment for clinically severe obesity and its associated co-morbidities^7^. Morbidly obese patients undergo a great metabolic improvement after bariatric surgery, even before weight loss has occurred^8^,^9^. In recent years, both clinical evidence and the development of animal models for bariatric surgeries, have contributed largely to the understanding of the underlying mechanism of metabolic improvement achieved after surgical intervention^10^,^11^, although the molecular mechanism has not completely been resolved.

Recently, epigenetics has been acquiring great importance and has been recognized as playing a significant role in complex diseases, providing mechanisms where by environmental factors can influence complex diseases such as obesity and type 2 diabetes (T2DM)^12^,^13^. Epigenetic modifications provide a potential molecular basis for the interaction between genes and environmental factors on glucose homeostasis and may also contribute to the development of insulin resistance/T2DM^14^. Also, some authors have shown that changes in DNA methylation could be a mechanism which contributes to the metabolic improvements after bariatric surgery^15^,^16^,^17^. Barres *et al*. showed that promoter methylation of peroxisome proliferator-activated receptor gamma coactivator-1αlpha (PPARGC1A) and pyruvate dehydrogenase kinase isozyme-4 (PDK4) were altered with obesity and were restored to non-obese levels after RYGB induced weight loss^16^. On the other hand, other studies have found an association between calorie restriction induced weight loss and changes in DNA methylation of specific gene promoters^18^,^19^. However, little is known about the changes in the methylation of the SCD gene promoter with bariatric surgery and its association with obesity and/or insulin resistance levels. Schwenk RW^20^ showed that the expression of SCD was lower in the liver of mice fed with a high fat diet when compared with animals fed with a high carbohydrate diet and correlated inversely with the degree of DNA methylation in the SCD promoter. Also, we recently showed changes in the DNA methylation of the SCD gene promoter after a nutritional intervention. Subjects who lost weight increased their levels of SCD methylation one year after the intervention^21^.

Since SCD seems to be related with obesity and insulin resistance, we aimed to study a) whether the methylation of the SCD gene promoter is associated with obesity and insulin resistance in morbidly obese subjects, b) its evolution after bariatric surgery and c) whether its change after bariatric surgery is associated with the changes found in different metabolic variables.

## Results

### SCD gene promoter methylation levels before and after RYGB

The characteristics of the control group and the morbidly obese subjects are shown in Table 1. As was expected, the morbidly obese subjects improved their metabolic, anthropometric and biochemical variables after RYGB (Table 1).

**Figure 1:**
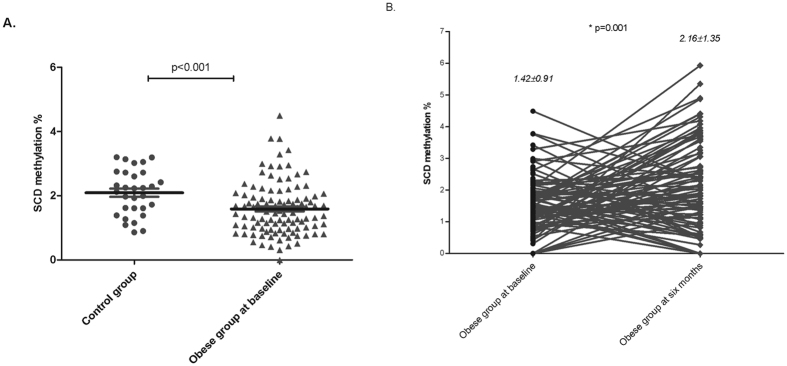
SCD methylation levels (%). (**A)** SCD methylation levels (%) in the control group and morbidly obese subjects before bariatric surgery. (**B)** SCD methylation levels (%) in morbidly obese subjects before and six months after bariatric surgery. Kruskall-Wallis test was used to compare means between control group and morbidly obese subjects before surgery. Wilcoxon test was performed to compare means between morbidly obese subjects before and six months after bariatric surgery.

DNA methylation levels of the SCD gene promoter in peripheral blood were lower in morbidly obese subjects before bariatric surgery (1.42 ± 0.91%) when compared with the control subjects (2.13 ± 0.68%) (Fig. 1A), but increased after RYGB to levels similar to those found in the control group (2.16 ± 1.35%). Figure 1B shows that there are some subjects that increase their DNA methylation levels (63.5% of morbidly obese subjects) whereas others present a decrease (36.5% of morbidly obese subjects). There were no significant differences in SCD DNA methylation levels between males and females; neither were there according to age.

### SCD mRNA expression and activity index

SCD mRNA expression was measured in peripheral blood mononuclear cells (PBMC) in a subset of morbidly obese subjects (n = 35) before and after RYGB. SCD mRNA expression was lower after RYGB compared to baseline levels, although these differences were not statistically significant (Table 1). Before and after RYGB, SCD mRNA expression significantly correlated with FFA levels (p = −0.464, r = 0.045, and p = 0.503, r = 0.039, respectively).

SCD activity indices were measured in the serum phospholipids fraction in a subset of morbidly obese subjects (n = 35) before and after RYGB, and in the control group (n = 30) (Table 1). SCD activity indices were significantly higher in morbidly obese subjects before RYGB compared to control subjects, decreasing significantly after RYGB to levels similar to the control group (Table 1).

No significant correlation was observed between SCD methylation levels and mRNA expression, neither with activity indices (Supplementary Table 1). Neither mRNA expression nor enzymatic activity were different between subjects who increased or decreased their SCD DNA methylation levels.

### SCD gene promoter methylation levels are associated with insulin resistance-related variables

There were no significant correlations between the SCD gene promoter methylation levels and anthropometric and biochemical variables in morbidly obese subjects, neither were there before or after RYGB (Supplementary Table 2).

**Figure 2:**
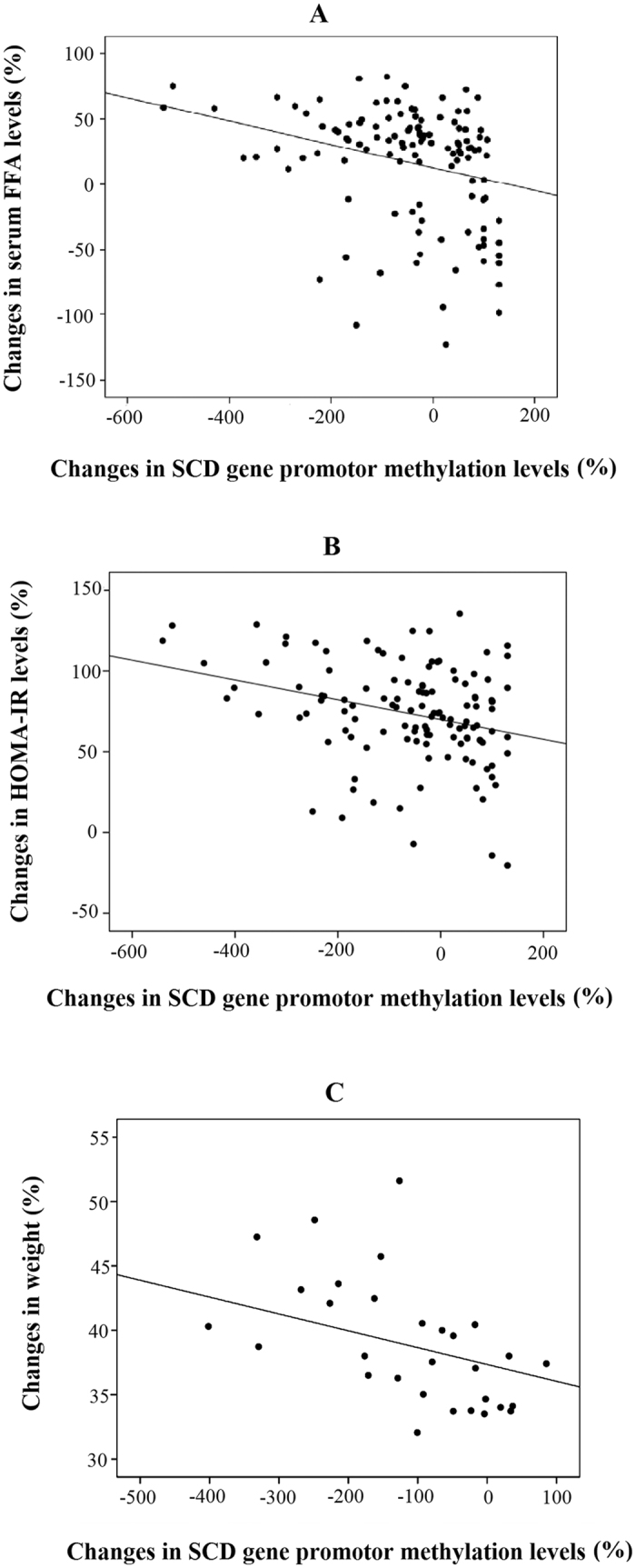
Association between the changes of SCD gene promoter methylation levels (%) with the change of serum free fatty acids level (FFA) (%) (**A**), and with the change of HOMA-IR level (%) (**B**) after RYGB; and with the change of weight (%) after RYGB in those morbidly obese subjects who lost a greater percentage of weight (above percentile 75th) (**C**).

To evaluate whether the changes observed in the anthropometric and biochemical variables in morbidly obese subjects after RYGB were related with changes in the DNA methylation levels of the SCD gene promoter, correlation tests were performed. A negative association was observed between changes in SCD gene promoter methylation and changes in free fatty acids (FFA) (r = −0.442, p = 0.006) (Fig. 2A) and HOMA-IR (r = −0.249, p = 0.035) (Fig. 2B). The increase of SCD gene promoter methylation levels was related to a decrease in FFA levels and in the insulin resistance index after RYGB. In fact, those morbidly obese subjects that increased the SCD gene promoter methylation decreased their free fatty acids levels after surgery (0.75 ± 0.17 mmol/L *vs* 0.53 ± 0.16 mmol/L). While, those that decreased the SCD gene promoter methylation did no change the free fatty acids levels (0.54 ± 0.19 mmol/L vs 0.51 ± 0.17 mmol/L). On the contrary, a positive association was found between changes in SCD gene promoter methylation levels and changes in adiponectin levels (r = 0.389, p = 0.019).

In those morbidly obese subjects who lost a greater percentage of weight (above percentile 75th) (n = 30), a significant association was found between the change of the SCD gene promoter methylation level and the change of weight (r = −0.585, p = 0.011) (Fig. 2C). In those morbidly obese subjects who lost a lower percentage of weight (below percentile 75th) (n = 90), the change of the SCD gene promoter methylation level was significantly associated with the change of FFA (r = −0.460, p = 0.010) and HOMA-IR levels (r = −0.335, p = 0.012).

### Predictor variables of SCD promoter methylation

To test which variables could explain the variation of the SCD gene promoter methylation levels after RYGB, a linear regression model was performed. We showed that FFA levels at baseline were significantly associated with SCD methylation level changes in a model adjusted for age, sex, weight, HOMA-IR and adiponectin levels at baseline. The 36% of the variability of SCD is explained by FFA or other covariables (Table 2).

## Discussion

The main finding of our study was that SCD gene promoter methylation levels were decreased in morbidly obese subjects before bariatric surgery, raising six months after RYGB to similar levels to those found in a group of subjects with similar BMI. This increase was associated with changes of insulin resistance related metabolic parameters after RYGB. Of special relevance are the inverse relationships between the DNA methylation of the SCD promoter and FFA and HOMA-IR, and the direct association with adiponectin levels.

SCD is the enzyme that catalyses the conversion of saturated fatty acids to MUFA. Animals and human studies have shown the association between SCD and obesity and related metabolic disorders, such as insulin resistance and inflammation^22^. These pathologies are clearly improved after bariatric surgery. There are numerous studies explaining the possible mechanisms involved in the metabolic improvement after bariatric surgery^23^,^24^. Recently, some authors have shown that changes in DNA methylation could be another mechanism to take into account in this improvement. Our results show that RYGB produces a significant increase in SCD methylation promoter levels. In this context, Barres *et al*. observed that promoter-specific DNA methylation of PGC-1α and PDK4 genes is modified after RYGB in skeletal muscle^16^, being restored to non-obese levels after RYGB-induced weight loss. Kirchner *et al*. showed an increase of methylation levels in whole blood in PDK4, interleukin 1B, interleukin 6 and tumor necrosis factor (TNF) promoters one year after RYGB^15^. These authors proved that RYGB induced more epigenetic changes than only a very low calorie diet. Nilsson *et al*., also demonstrated small changes in the DNA methylation of 51 gene promoters from whole blood after RYGB (at six months), being similar to a control group^25^. Finally, Benton *et al*. found differential methylation within genes associated with obesity, epigenetic regulation and development in human adipose tissue from obese women before and after a gastric bypass^17^. So, surgery induced mechanisms could modify the DNA methylation of specific genes, and in this context, DNA methylation may play an important role in the metabolic improvement after bariatric surgery. However, the potential mechanisms are still unknown.

Epigenetic modifications are regulated by numerous nutritional and environmental factors^26^. In this regard, the changes found in the biochemical parameters after RYGB may influence DNA methylation. For example, Barres *et al*. provided evidence that elevated levels of FFA induce acute hypermethylation of the PGC-1α and mitochondrial transcription factor A (TFAM) promoters^27^. However, in our study, we found a strong negative relationship between changes in FFA and SCD methylation levels. Furthermore, we observed as although the overall change was small, some subjects increased their levels of SCD DNA methylation whereas some others presented the opposite effect, showing a variable response between patients after surgery. This different effect could be associated with the changes in FFA levels. However, this different response was not associated with the changes in SCD mRNA expression and activity indices. It could be possible that SCD methylation promoter levels are influenced by FFA levels. We could hypothesize that the higher FFA levels found before RYGB could produce a hypomethylation of the SCD promoter by unknown mechanisms.

SCD is regulated, among other factors, by fatty acids. SCD mRNA levels in 3T3-L1 adipocytes were repressed by 80% of the original levels when these cells were exposed to 300 μM arachidonic acid^28^. DNA methylation could be another factor to take into account in the regulation of SCD expression. Concerning this, a previous study showed that the methylation of lysine 3 of histone H9 (H3K9) was greatly promoted by n-3 polyunsaturated fatty acids in obesity^29^. The methylation of H3K9 has been associated with transcriptional silencing^30^. A recent study has shown that the treatment with palmitate for 48 h affects genome-wide mRNA expression and DNA methylation patterns in human pancreatic islets^31^. As in a previous study of our group performed in adipose tissue, we have also found a negative association between mRNA expression in PBMC and FFA levels^4^. However, we have not found significant association between SCD mRNA expression and SCD methylation levels. It is possible that FFA can decrease DNA methylation and mRNA expression by different unknown mechanisms.

Furthermore, FFA might modify DNA methylation in other CpG sites of the SCD gene that have not been analyzed in our study, thus affecting mRNA expression levels of SCD. In this study, we only analyse 8 CpG in the SCD promoter region. This requires more studies about the effect of different fatty acids on the SCD methylation promoter levels^32^.

As in our previous study, serum SCD activity indices were not associated with SCD mRNA expression in PBMC, neither with SCD methylation levels^4^. This could be explained because this measurement of SCD activity in serum mainly reflect the hepatic activity of SCD, since these indices are derived from fatty acids present in VLDL, LDL and HDL, which have their origin mainly in the liver, not in PBMC^33^

A limitation of our study is that we have not analyzed the whole SCD promoter. This region contains a high number of CpG sites and is also highly polymorphic, which makes the design more complicated. Anyway, the studied region has also been previously associated with metabolic parameters, reinforcing the results^21^. Furthermore, a recent article has also found changes in DNA methylation in three CpG sites close to the transcription start site of SCD according to sleep duration^34^. These sites were in the same region that we have analyzed. On the other hand, the observed changes were in a range very low. We are conscious of this limitation but in opposite, we performed several runs with duplicates and we have adjusted the technique for this assay^21^. We included unmethylated and methylated DNA as controls in each run to verify the reproducibility. On the other hand, some authors have shown that in several genes, a very small change in DNA methylation may alter the gene expression^35^.

Finally, our previous work performed in a prospective cohort intervention study with a control group, showed that the subjects who lost weight after a nutritional intervention had higher levels of SCD gene promoter methylation after the intervention^21^. The results of the present study are consistent with our previous ones. We observed a negative relationship between changes in SCD promoter methylation and changes in weight after RYGB, but only in those subjects who lost weight above the 75 percentile. We don’t know the reason for the presence of this association only in this group of morbidly obese subjects. It could be possible that other non-analyzed factors in this study could be involved in this relationship. After RYGB, there are extreme changes in the levels of numerous molecules. Perhaps, any of these factors or molecules could be related to this association in a situation such as extreme obesity, and not in a less severe morbid obesity.

## Conclusion

In conclusion, we found that RYGB produces an increase in the low SCD methylation promoter levels found in morbidly obese subjects. This modification of methylation levels is associated with changes found in HOMA-IR, adiponectin, and above all in FFA levels. These variables are closely linked to insulin resistance state. But in those morbidly obese subjects with a higher weight change, SCD methylation promoter levels are associated with weight changes. More studies are needed in order to understand the association between gene methylation levels and obesity and insulin resistance state.

## Methods

### Subjects

The study included 120 subjects with morbid obesity who underwent laparoscopic Roux-en Y gastric by-pass (RYGB) and 30 obese subjects with a similar body mass index (BMI), HOMA-IR, age and percentages of males/females as the morbidly obese subjects six months after RYGB. Morbidly obese subjects were studied before and 6 months after RYGB. Subjects were excluded if they had cardiovascular disease, arthritis, acute inflammatory disease and infectious disease. Samples were processed and frozen immediately after their reception at the Regional University Hospital Biobank (Andalusian Public Health System Biobank). The research was carried out in accordance with the Declaration of Helsinki (2008) of the Word Medical Association. All the participants gave their written informed consent and the study was reviewed and approved by the Ethics and Research Committee of the Regional University Hospital, Malaga, Spain.

### Laboratory measurements

Blood samples were collected after a 12-hour fast. The serum was separated and immediately frozen at −80 °C. Serum biochemical variables were measured in duplicate as previously described^36^,^37^. The homeostasis model assessment of insulin resistance (HOMA-IR) was calculated: HOMA-IR = fasting insulin (μIU/mL) × fasting glucose (mmol/L)/22.5.

### Isolation of PBMC

Blood samples were obtained after a 12-h fast. PBMCs were only isolated from a subset of morbidly obese subjects before and after RYGB (n = 35). PBMC were isolated by Ficoll standard density gradient centrifugation^37^.

### Real-time quantitative PCR

Total RNA from PBMC was prepared using Trizol reagent (Gibco BRL Life Technologies, Carlsbad, CA) according to the manufacturer’s instructions. Complementary DNA was obtained and quantified as previously described^37^. The primers for the PCR (Sigma-Proligo, St Louis, MO) were as follows: SCD (NM_005063): Fwd: 5′-cagtgtgttcgttgccactt-3′, Rev: 5′-ggtagttgtggaagccctca-3′, β-Actin (NM_001101): Fwd: 5′-tacagcttcaccaccacggc-3′, Rev: 5′-aaggaaggctggaagagtgc-3′. The cycle threshold (Ct) value for each sample was normalized with the expression of β-actin (housekeeping gene). Calculation of relative expression levels of the different transcripts was performed based on the cycle threshold (CT) method. CT values from each experimental sample were then used to calculate mRNA gene expression levels, which were expressed as the percentage of relative gene expression.

### SCD activity index in serum

SCD activity index in serum were only analyzed from a subset of morbidly obese subjects before and after RYGB (n = 35) and in control subjects (n = 30). Total lipids from serum were extracted with chloroform-methanol 2:1 (v/v). The phospholipids were separated by thin-layer chromatography on silica gel plates (Merck, Darmstadt, Germany) with hexane-ethylic ether-acetic acid (80:20:2, v/v/v) as the developing solvent. The fatty acid composition of phospholipids was analyzed as described^38^. SCD activity indices were calculated from phospholipids composition as the ratio of 18:1(n-9)/18:0 and 16:1(n-7)/16:0 fatty acids.

### DNA isolation

Venous blood samples were collected from all subjects and genomic DNA was isolated from peripheral blood using QIAmp DNA Blood Kit (Qiagen, Hilden, Germany) in a QIACUBE instrument (Qiagen) according to the manufacturer’s recommended protocols. DNA samples were stored at −20 °C.

### DNA methylation analysis by pyrosequencing

DNA methylation analyses were performed on bisulfite-treated DNA using highly-quantitative analysis based on PCR-pyrosequencing. The bisulfite conversion was performed with 500 ng genomic DNA isolated from peripheral blood using the EpiTect bisulfite kit (QIAGEN) as recommended by the manufacturer. The PyroMark™Q96 ID Pyrosequencing System (QIAGEN) was used to determine the methylation status of the CpG island region of the SCD gene promoter.

For the methylation analysis of the SCD gene promoter (NM_005063.4) we used the primers and PCR conditions previously published^21^, the primer sequences were designed using the MethPrimer software^39^ in a region at −400 bp from the transcriptional start site (TSS), including 8 CpG dinucleotides. The target region and its location are illustrated in Supplementary Figure 1.

The degree of methylation was expressed for each DNA locus as the percentage of methylated cytosine over the sum of methylated and unmethylated cytosines. Non-CpG cytosine residues were used as built-in controls to verify bisulfite conversion. Correlation between methylation at all eight CpG sites was high (p < 0.001), therefore the mean for all the sites was expressed as %5 mC. The values are expressed as the mean for all the sites. We also included unmethylated and methylated DNA as controls in each run (New England Biolabs).

### Statistical analysis

The continuous variables are shown as the mean and standard deviation and the classification variables as proportions. Normal distribution was tested by the Kolmogorov-Smirnov test. The Wilcoxon paired-sample and the Kruskall-Wallis tests were used for intra-group and inter-group comparisons, respectively. Correlation analyses were performed with the Spearman correlation coefficient. Multiple linear regression models were used to evaluate the predictive capacity of different metabolic parameters on SCD gene promoter methylation changes. The models were adjusted for possible confounding variables such as age, gender and body mass index (BMI) at baseline. Changes in the variables due to RYGB were expressed as percentages and were calculated as (variable_baseline_ − variable_at 6 months_) × 100/variable_baseline_. All analyses were performed using R statistical software, version 2.8.1 (Department of Statistics, University of Auckland, Auckland, NZ; http://www.r-project.org/)^40^.

## Additional Information

**How to cite this article**: Morcillo, S. *et al*. Changes in SCD gene DNA methylation after bariatric surgery in morbidly obese patients are associated with free fatty acids. *Sci. Rep.* **7**, 46292; doi: 10.1038/srep46292 (2017).

**Publisher's note:** Springer Nature remains neutral with regard to jurisdictional claims in published maps and institutional affiliations.

## Supplementary Material

Supplementary Material

## Figures and Tables

**Table 1:** Characteristics of the study subjects.

	Control group	MO subjects before RYGB	MO subjects six months after RYGB
Age (y)	47.2 ± 5.8	43.2 ± 9.4	—
Sex (Male/female) (%)	32.1/67.9	30.2/69.8	—
Weight (Kg)	85.1 ± 11.7	140.2 ± 25.1^2^	96.2 ± 19.6^b^
Waist (cm)	110.8 ± 8.1	137.3 ± 16.4^2^	108.8 ± 13.1^b^
BMI (kg/m^2^)	33.6 ± 2.3	50.9 ± 7.1^2^	35.1 ± 6.6^b^
Glucose (mg/dl)	103.2 ± 12.5	116.3 ± 46.9	83.6 ± 10.6^b^
Insulin (μIU/ml)	10.2 ± 4.9	21.2 ± 9.3^2^	9.9 ± 3.8^b^
HOMA-IR	2.6 ± 1.5	6.2 ± 4.1^2^	2.1 ± 0.9^b^
FFA (mmol/L)	0.50 ± 0.24	0.63 ± 0.21^1^	0.52 ± 0.17^a^
Adiponectin (μg/ml)	8.7 ± 3.9	8.2 ± 3.5	11.8 ± 8.7^a^
Leptin (ng/ml)	15.4 ± 9.2	122.1 ± 86.1^2^	30.9 ± 31.4^b^
Activity index 16:1(n-7)/16:0	0.015 ± 0.007	0.079 ± 0.12^1^	0.019 ± 0.02^b^
Activity index 18:1(n-9)/18:0	0.654 ± 0.13	1.72 ± 2.7^1^	0.719 ± 0.23^a^
SCD mRNA expression levels	—	2.17 ± 2.5	1.56 ± 1.07

MO: morbidly obese. BMI: Body mass index. FFA: free fatty acids. RYGB: Roux-en Y gastric bypass.

^1^p < 0.01, ^2^p < 0.001: Significant differences between morbidly obese subjects before RYGB and control group.

^a^p < 0.01, ^b^p < 0.001: Significant differences in the morbidly obese subjects between before and six months after RYGB.

**Table 2:** Baseline predictor variables of SCD promoter methylation changes found in morbidly obese subjects after RYGB.

	Standardized β	P value
	R^2^ = 0,326	
Sex (males/females)	−0,370	0,075
Age (years)	0,281	0,120
Weight (Kg)	−0,383	0,095
FFA(mmol/L)	−0,434	0,020
HOMA-IR	0.026	0.884
Adiponectin (μg/ml)	0.051	0.752

FFA: free fatty acids.
