# Supplementary material for: Changes in SCD gene DNA methylation after bariatric surgery in morbidly obese patients are associated with free fatty acids
Source: Sci Rep. 2017 Apr 10;7:46292. doi: 10.1038/srep46292 (PMC5385880; doi:10.1038/srep46292)

Title: Changes in SCD gene DNA methylation after bariatric surgery in morbidly obese patients are associated with free fatty acids

Authors: Sonsoles Morcillo*[1,2], Gracia Mª Martín-Núñez*[1], Sara García-Serrano[3,4], Carolina Gutierrez-Repiso[1], Francisca Rodriguez-Pacheco[3], Sergio Valdes[3,4], Montserrat Gonzalo[3], Gemma Rojo-Martinez[3,4], Francisco J. Moreno-Ruiz[5], Alberto Rodriguez-Cañete[5], Francisco Tinahones[1,2], Eduardo García-Fuentes[2,6].

[1]*Unidad de Gestión Clínica de Endocrinología y Nutrición, Instituto de Investigación Biomédica de Málaga (IBIMA), Hospital Clínico Virgen de la Victoria, Málaga, Spain.*

[2]*CIBEROBN, Instituto de Salud Carlos III, Málaga, Spain.*

[3]*Unidad de Gestión Clínica de Endocrinología y Nutrición, Instituto de Investigación Biomédica de Málaga (IBIMA), Hospital Regional Universitario, Málaga, Spain.*

[4]*CIBERDEM, Instituto de Salud Carlos III, Málaga, Spain.*

[5]*Unidad de Gestión Clínica de Cirugía General, Digestiva y Trasplantes, Instituto de Investigación Biomédica de Málaga (IBIMA), Hospital Regional Universitario, Málaga, Spain.*

[6]*Unidad de Gestión Clínica de Aparato Digestivo, Instituto de Investigación Biomédica de Málaga (IBIMA), Hospital Clínico Virgen de la Victoria, Málaga, Spain.*

** These authors contributed equally to this work*

Supplementary Table 1: Correlations between SCD methylation levels (%) and mRNA expression and activity of SCD

	At baseline					
	**16:1n7 / 16:0**		**18:1(n9)/18:0**		**mRNA SCD**	
	r	P value	r	P value	r	P value
**SCD methylation levels at baseline**	-0.367	0.050	-0.310	0.102	0.084	0.742
	At six months after surgery					
	**16:1n7 / 16:0**		**18:1(n9)/18:0**		**mRNA SCD**	
	r	P value	r	P value	r	P value
**SCD methylation levels after surgery**	0.143	0.583	0.416	0.096	-0.180	0.400

Supplementary Table 2: Correlations between anthropometric and biochemical variables, and SCD methylation levels.

	SCD meth (%) at baseline[1]		SCD meth (%) at six months[2]	
	r	P value	r	P value
**Weight (Kg)**	-0,068	0,476	0,117	0,277
**Waist (cm)**	-0,099	0,327	0,003	0,974
**BMI (kg/m²)**	-0,174	0,067	0,035	0,749
**Glucose (mg/dl)**	0,120	0,201	-0,134	0,206
**Insulin (µIU/ml)**	0,041	0,655	-0,168	0,112
**Total cholesterol (mg/dL)**	0,117	0,214	-0,075	0,486
**Triglycerides(mg/dL)**	0,111	0,241	-0,072	0,502
**HDL(mg/dl)**	0,187	0,050	-0,123	0,251
**HOMA-IR**	0,105	0,267	-0,137	0,202
**FFA (mmol/L)**	-0,060	0,641	-0,137	0,344
**Adiponectin (µg/ml)**	0,161	0,227	-0,065	0,676
**Leptin (ng/ml)**	0,132	0,337	0,173	0,260

SCD methylation levels are expressed as the mean of all 8 sites CpG (%)

[1] Correlations were performed between baseline SCD methylation levels and their corresponding baseline variables

[2] Correlations were performed between SCD methylation levels after surgery and their corresponding variables at six months.

Supplementary Figure 1

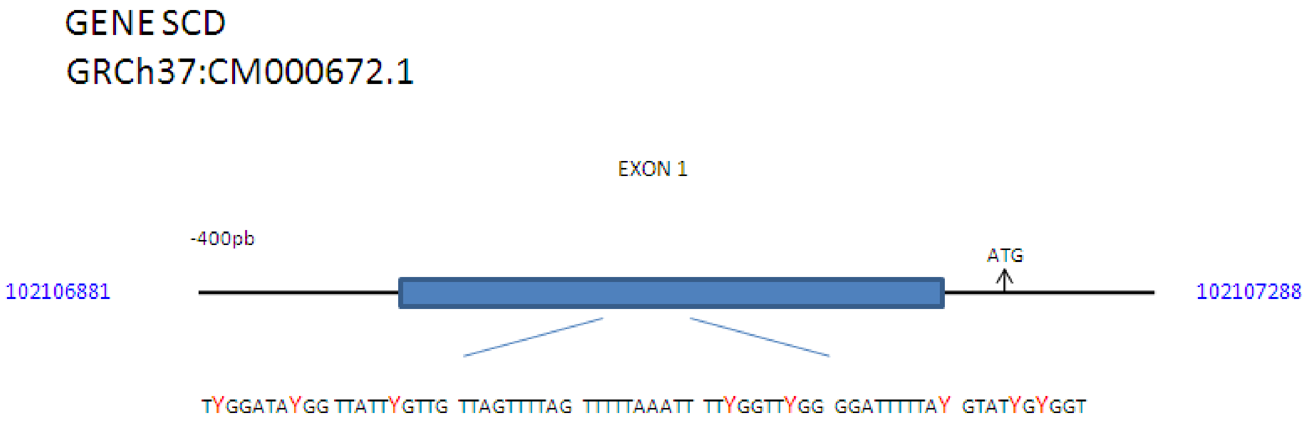

Supplement: Supplementary Material [file srep46292-s1.pdf]
